# Experiences and meaning of loneliness beyond age and group identity

**DOI:** 10.1111/1467-9566.13539

**Published:** 2022-09-08

**Authors:** Melina Aikaterini Malli, Sara Ryan, Jane Maddison, Kalpa Kharicha

**Affiliations:** ^1^ Department of Social Care and Social Work Manchester Metropolitan University Manchester UK; ^2^ The Oxford Institute of Population Ageing University of Oxford Oxford UK; ^3^ School for Business and Society University of York York UK; ^4^ NIHR Health & Social Care Workforce Research Unit King’s College London London UK

**Keywords:** loneliness, qualitative research, symbolic interactionsism

## Abstract

Research into loneliness has focussed on subpopulations, and in particular those defined by age, identifying specific contextual factors contributing to their experiences. We suggest that the ‘essence’ of loneliness cannot be fully captured by examining a unitary group and argue for broader and diverse sampling to better understand how loneliness is experienced. Informed by a symbolic interactionist approach, this study aims to elucidate experiences and meaning of loneliness among a heterogeneous group of adults. In depth interviews were conducted with a diverse sample of 37 individuals, aged 18–71 years who had experienced loneliness in the UK. Using thematic analysis, four themes were identified: Loneliness as lacking, loneliness as abandonment, lingering loneliness and the unspoken and trivialised experience of loneliness. Our analysis signals the complexity of loneliness  did not necessarily conform to one‐dimensional conceptualisations of the phenomenon. Loneliness is linked to interpersonal relationships, but also associated with participants’ roles and identity within society. Thus, society exacerbates and creates loneliness. Implications for the support and provision of loneliness are also discussed.

## INTRODUCTION

The 21st century has been characterised as the era of loneliness. It has been referred to as a ‘silent plague’ by the media and described as the ‘widespread disorder of our times’ (Verhaeghe, 2015, p. 205). Globally, surveys indicate an unprecedented rise in loneliness with around a third of people affected and one in twelve affected severely (Cacioppo & Cacioppo, 2018). It is viewed as a health crisis, associated with a plethora of deleterious health outcomes comparable to obesity and smoking and used as a trope to illustrate the failing of modern industrialised times (Vincent, 2020). In the UK, the topic was moved to the political arena and the first Minister of Loneliness was appointed by the British Conservative government in January 2018. Furthermore, the former Prime Minister Teresa May stated that General Practitioners (GPs) will be able to refer people experiencing loneliness to community groups and services as part of social prescribing (Department for HM Government, 2018).

While there is no consensus about the definition of loneliness, three theoretical frameworks have been offered in the study of the phenomenon: the existential, the social needs and the cognitive perspectives. The first conceives loneliness as a natural and necessary component of human existence (Moustakas, 2016). The social needs perspective views loneliness as a result of the absence of needed relationships and lack of interpersonal affirmation with roots in attachment theory and childhood. Weiss (1989), the pioneer of this theory, made a distinction between two forms of loneliness: emotional and social. The former is the result of a lack of close, intimate attachment to another person. Thus, one views their social relations as qualitatively lacking. The latter reflects the absence of a social support system; thus, one perceives their network of social relationships to be quantitatively inefficient. Finally, the cognitive perspective describes loneliness as a negative and involuntary experience that arises from the perceived discrepancy between desired and actual emotional and/or social relationships (Perlman & Peplau, 1982). The latter definition is most commonly used in research and has served to create interventions that aim to modify social cognition (Masi et al., 2011). It also distinguishes different forms of loneliness based on their chronicity. While transient loneliness may arise due to circumstances (e.g., moving geographically or retirement) and people affected by it tend to adjust to their environment, chronic loneliness alludes to feelings that last for more than 2 years and are hard to change or an intense feeling that is hard to endure (Young, 1982). It has been attributed of the inability to develop satisfying social relationships over the years. It has been associated with social deficits and people who live alone (Shiovitz‐Ezra & Ayalon, 2010). It should be noted, however, that the term ‘chronic’ alludes to the pathologisation of long‐term loneliness and it has therefore been challenged by scholars who have preferred, as we do, to use the terms persistent or prolonged loneliness (Victor et al., 2018). Common ground in these three approaches is that loneliness is an emotionally negative experience that increases the risk of adverse effects in physical and mental health.

A limitation of research in this area is that it has mostly focussed on generating a quantifiable definition of the term to measure its antecedents and physical and mental health consequences. Furthermore, the prevalence of loneliness in later life has not changed for decades, notwithstanding the attention on the subject and host of interventions (Barreto et al., 2021). We suggest that more qualitative and conceptual/theoretical work is needed to better understand the phenomenon in order to develop appropriate responses. Furthermore, studies have mainly explored deficits in interpersonal relationships that cause loneliness with an emphasis on micro‐level factors and the individual (e.g., personality, social skills, demographics, resources and physical mobility) whilst there is less research exploring the role of communities and societal relationships that contribute to loneliness (Wong et al., 2017). The multidimensional nature of loneliness has been characterised as a highly subjective and idiographic experience to the extent that Weiss (1989) suggests that it is an elusive phenomenon that can better be described than defined. Nevertheless, there is an inherent and largely underlying assumption that the concept of loneliness is a common construct with a universally understood meaning that can be apprehended by quantitative methods such as the UCLA Loneliness Scale (Russell, 1996) and the de Jong Gierveld loneliness scale (Gierveld & Van Tilburg, 2010).

The polymorphic nature of loneliness, however, is difficult to capture and its essence is hard to apprehend using only quantitative measures. Its definition is further complicated by closely related concepts such as ‘social isolation’, often used interchangeably in everyday discourse. In contrast to loneliness, however, social isolation is a quantifiable concept and considered to be an objective state. Loneliness is based on individualistic appraisal and can occur in the presence of company as it can be characterised by the lack of ‘meaningful’ social relationships rather than the magnitude of a person’s social network (Tanskanen & Anttila, 2016). Indeed, studies suggest that although a lack of social contacts can lead to loneliness, it is possible to live a solitary life and not feel lonely. Being alone may be an enjoyable experience that can lead to personality development and creative activity (Galanaki et al., 2015). Reviews of intervention studies (e.g., Victor et al., 2018), draw attention to insufficient separation of related concepts for underpinning the persistent difficulty in establishing ‘what works’ in tackling loneliness, or social isolation, for whom and in which circumstances.

Furthermore, although there is a wealth of research exploring experiences of loneliness and the meaning for those who describe it, loneliness has been most commonly examined in relation to specific segments of the population. Advanced age and ageing are often equated with becoming lonely: indicatively, Mansfield et al.’s (2021) recent synthesis of qualitative studies exploring loneliness up to 2018 indicated that more than half of these studies focussed on ageing. Although there has also recently been an interest in younger people and loneliness (Osborn et al., 2021), there is a need to explore the concept beyond the constraints of age. Other qualitative studies explore the concept of loneliness in relation to specific groups, such as disabled people or people with mental ill health, again identifying explicit contextual and person‐related factors contributing to the experience of loneliness (Karhe & Kaunonen, 2015; Lindgren et al., 2014). Although there is a sociological merit and a need to consider the concept of loneliness among groups of people with specific identities, loneliness is a condition of human existence and there is a need to better understand the meaning of it through a broader population that might have a greater diversity of perspectives regarding the processes that define it.

Our research, similar to Neves et al. (2019), that aimed to understand the meaning frail older people in institutionalised settings in Australia ascribed to their lived experiences of loneliness, was informed by a symbolic interactionist approach. Thus, we were interested in understanding how participants construct and negotiate the meaning of their experiences of loneliness. These meanings are modified through an interpretive process by participants and arise through interaction with others (Blumer, 1986, p. 72). Our aim was to generate a deeper understanding of the experiences and meaning of loneliness through an analysis of in‐depth interview data. Blumer strongly believed that research methods should be faithful to the empirical world under investigation. Following this fidelity, we did not provide a pre‐existing theoretical framework of what loneliness may be, allowing space for subjective constructions of loneliness to emerge during interviews that could further elucidate the meaning of the experience. Instead of definitions of loneliness, we used sensitising concepts such as song lyrics and popular fiction as heuristic devices to help participants formulate their sense making. Blumer’s version of naturalism and the accompanying avoidance of precise specification (Atkinson & Housley, 2003) was particularly relevant, given the lack of consensus around the definition of loneliness and the emotional layers to it. From a symbolic interactionist perspective, ‘emotions are not merely natural impulses. Rather, they are shaped by both culture (…) and our human capacity to react and make sense of our feelings’ (Fields et al., 2006, p. 156). Participants actively engaged in meaning making during the process of the interviews as they were encouraged to explore and consider their experiences through careful probing and questioning by the first author. In constructing these meanings, participants drew on ‘symbolic emotional resources’ (Sawicka, 2017), such as stories and metaphors, and it is to our methods we now turn.

## OBJECTIVE OF THE STUDY

The aim of the study was to gain a deeper understanding of the experiences and meaning of loneliness by attending to the perceptions of those who identify as lonely or had experienced loneliness, and so moving beyond loneliness expressed by a unitary group. Thus, the objective was to describe, interpret and provide a comprehensive insight into personal experiences of the phenomena and to capture the meaning of loneliness through the narratives of a diverse range of voices and several population groups whose experiences have been neglected in research.

## METHOD

A qualitative cross‐sectional study employing in‐depth interviews was used to understand how participants made sense of the experiences of loneliness and the meaning they attributed to it. This was part of a wider NIHR‐funded study exploring whether a co‐design intervention used successfully in health care could be used in social care settings.

### Sample and recruitment

A purposive sampling framework was devised to recruit a ‘maximum variation sample’ (Coyne, 1997) to meaningfully include overlooked groups and marginalised populations and generate a diversity of perspectives and experiences.

Participants were recruited in various ways, including mental health charity advertisements, local authority newsletters, personal contacts, snowballing through existing contacts and social media platforms. More specifically Facebook, which has been identified as a useful tool in approaching seldom heard populations (Parkinson & Bromfield, 2013) was used. Flyers of the study were posted on relevant public Facebook pages but also closed support groups. Due to the strict conditions of access of the latter and in respect to the privacy of the users, the study was only advertised after permission was granted by the administrators through private messages providing details of the research (Brickman Bhutta, 2012). More than 30 closed Facebook groups were approached relating to mental illness, disabilities, drug and alcohol dependencies, and LGBTQ communities of which three declined to advertise the study. Participants had to be 18 years old or over and have experienced loneliness. Although some studies have avoided using the term ‘loneliness’ to recruit participants due to negative connotations of the word (Victor et al., 2000), it was clearly stated in our flyers so as not to mislead potential participants about the topic of our study. All participants received a £30 voucher in recognition of their time and contribution to the research.

Participant recruitment ceased when data were saturated, thus, when the new data repeated what was expressed in previous interviews without bringing new contribution to the understanding of the phenomenon (Hennink & Kaiser, 2021). The sample included 37 individuals, aged 18–71 years, 13 identifying as male, 23 as female and 1 as non‐binary. A further two people were interviewed but later withdrew.

Our sample constitutes a heterogeneous group even within specific categories. Most participants described performing multiple intersecting identities and had diverse life experiences. Many identified with multiple minority groups based on their disability status, mental health status, immigration status, race, sexuality and socioeconomic status. Due to this multiplicity of identity and intersectionality of the participants, there was heterogeneity within each social group. In line with intersectionality theories (Stewart & McDermott, 2004), multiple identities produce new forms of experiences that cannot simply be reduced to the original identities that went into them. Accordingly, disaggregating unitary identities would require discarding the interactivity of social identity structures and omitting unique realities. Therefore, although in Table 1 we outline the number of participants that identified with some marginalised identities, each category was not explored separately.

**TABLE 1:** Identities and roles participants identified to

Identities	Number of participants
Individuals with physical disabilities	3
Individuals with mental illness	21
Individuals with learning disabilities	2
Autistic people	4
Individuals in bereavement	4
People from the LGBTQ community (lesbian, gay, bisexual, transgender and queer)	5
Migrants	6
Substance users	4
People living with HIV	4
Individuals who have experienced domestic violence	4
Unpaid carers	2
Black and Minority Ethnic (BME) people	3
People who have experienced homelessness	3

A key output for the study was the production of a module on loneliness on SocialCareTalk.org, an online resource developed for members of the public and for learning and teaching social care students and professionals.

### Procedure

The study was reviewed and granted an ethically favourable opinion by the NHS Research Authority Social Care Ethics Committee (October 2020, 20/WM/0223). All participants were given a detailed information sheet and were encouraged to seek clarifications and ask questions in relation to the study and the research process. Participants were interviewed between October 2020 and January 2021.

Thirty of the interviews were conducted online using Microsoft Teams and seven participants preferred the telephone. None were conducted face to face due to COVID‐19 restrictions. A warmup discussion preceded the interview to make the participants feel at ease. This is especially pertinent to sensitive and stigmatising topics, as is loneliness. The interview was divided into two sections. It started with an open‐ended question (‘Can you tell me a little bit about yourself and how loneliness came into your life?’) with minimal interviewer structuring. In this part of the interview, participants were given a chance to present a narrative based on their own lived experience. The second part was based on a topic guide grounded on available literature covering loneliness and support, identity, and loneliness and the media. To bridge the two parts of the interview, the researcher started with immanent questions, that is, questions about topics raised by the participant in their primary narrative. Subsequently, exmanent questions, thus aspects that had not been mentioned by the participant but belonged to the sphere of the researcher’s interest were asked. In this article, our analysis focuses on how participants made sense of the experiences of loneliness. It should be highlighted that the researcher refrained from providing a definition for loneliness as we wanted to understand how people articulated and described their experiences of loneliness. More accessible interview schedules were developed for people with learning disabilities. A small Public and Patient Involvement (PPI) group helped assess the appropriateness and wording of the interview schedule and adapt the language and structure.

The interviews lasted between 45 min and 2 h and were recorded with participants’ permission, transcribed verbatim by freelance transcribers and checked by the first author. The transcript was returned to individuals to allow them the opportunity to review their data before data analysis. Only one participant changed some wording on their transcript.

One interview was conducted in Greek by the first author who is bilingual and translated.

Effort was taken to ensure the relationship between interviewer and participant would not be hierarchical and exploitative, and the researcher worked on a ‘participatory model’ in which she shared elements of her own biography with participants. However, it is acknowledged that loneliness in relation to this population was explored through an outsider’s viewpoint.

### Analysis

A multi‐stage inductive thematic analysis was carried out as a systematic method of generating themes and patterns within the data following Braun and Clarke (2006) guidelines and using the organisational support of NVivo 12. Initially, the data were read carefully by the first author in a process of familiarisation before it was coded (using ‘nvivo’) to maintain participants’ meaning as far as possible. The descriptive codes were organised into categories and these categories were subsequently analysed conceptually in what Braun and Clarke (2006, p. 84) describe as a process of ‘latent analysis’ to examine underlying ideas, assumptions, connections and links within the data. From this analytic stage, themes were identified and the data were systematically reviewed to ensure that a name and clear definition for each theme were produced and that these themes worked in relation to the coded extracts. To achieve trustworthiness, the second and third author independently coded seven randomly selected transcripts and a high level of intercoder agreement was reached. To increase the integrity and trustworthiness of the study, the first author kept a reflective diary during the interviews, transcription and analysis phase, to evaluate how subjective and intersubjective elements influence the research process (Finlay, 2002).

## RESULTS

Four themes were identified which capture participants’ experiences of loneliness: loneliness as lacking, loneliness as abandonment, lingering loneliness and the unspoken and trivialised experience of loneliness. Importantly, these themes apply across the different categories of participants rather than being associated with a particular marginalised identity. There is also evidence in the data of the use of isolation and loneliness as synonyms in everyday language.

The delineated themes are presented and accompanied by substantive anonymous quotes from the participants which have been unaltered to maintain the participants’ authenticity.

### Loneliness as lacking

Loneliness was linked to the loss of important and close relationships but was also experienced as the absence of close relationships with people who could genuinely understand them, empathise with them and affirm their importance.

#### Loneliness as loss

Participants referred to various experiences of loss. Bereavement (past or recent) and subsequently loneliness was a central element in their narratives as seen in previous studies (Bennet & Victor, 2012). As the demographic of our participants does not consist solely of older people where loss of loved ones could be perceived a natural part of the ageing process, the death of beloved people in most cases were unanticipated and accompanied by emotional trauma. The untimely passing of their loved ones did not only deprive participants of their company and affection, but also meant the loss of an envisioned future together. P29 who lost her partner abruptly in her late 30s explained:

I know this woman who lost her husband, but she was in her 70s or 80’s. And I always say, “You lost a lot of past, but not so much future”. “I lost a lot of future and not so much past”. It’s very different to be on your own when you’re let’s say in your 70s and maybe you are a grandparent, and you have a completely different role in life than when you are just a young person who had their whole life to go.

(P29)

Irreplaceable bonds were described that could not be substituted, exacerbating loneliness and highlighting the loss. P20 mentioned in relation to the bereavement of her brother:

It’s strange because I come home from work and like I go to speak with him, or I go to phone him, or I go for like messaging about something. He’s not here

(P20)

Some discussed the inability, hesitation or even unwillingness of people generally to approach the grief of premature death. Thus, participants lacked the comforting and supportive responses that typical ‘mourners’ would receive and this inability to find solace perpetuated their loneliness. P40 said in relation to her brother’s death:

When your 27‐year‐old brother dies from a heroin overdose it’s like there’s no Hallmark card for that…people also don’t wanna talk about it because it’s a bit like “oh do we bring it up?” whereas people seem more comfortable bringing it up sort of if a parent or grandparents died.

(P40)

#### Loneliness as absence

For many participants, loneliness was not marked by the loss but by the absence of a meaningful other, a partner or a friend, to turn to and call on, a relationship that they never experienced. Many yearned for a connection with someone who they could talk to, confide in, discuss their problems with and ultimately share their world with. The following extracts illustrate this absence:

I have no‐one to share my life with… I don’t have someone to turn to, to tell my problems to, or anything like that.

(P37)

There’s moments when I’d like to sort of really connect with someone or when I’m feeling low, I want to sort of, because I don’t really have [um] many friends and [um] I think that’s when it does appear like [um] I feel that intense loneliness when I feel quite isolated.

(P1)

Even if participants perceived themselves as sociable, surrounded by peripheral and casual friendships, they described longing for emotional intimacy and the affection, nurturance and affirmation which was absent from their life. They lacked a companion who they could reveal their fears and thoughts to. Their self‐disclosure would be received in turn with validation, understanding and care. As P2 mentioned:

I suppose I would really like my friendship to be like quite close, friendships should be quite close [um] so I want to feel like, like family

(P2)

### Loneliness as abandonment

For many participants, loneliness began with strained relationships within their families, feelings of invisibility and abandonment from a very young age and experiences or fear of abuse. Others described feeling abandoned and excluded by society. Insufficient care, cuts in funding and what they felt was an invalidated identity restricted their social participation and imposed them to invisibility and feelings of abandonment.

#### Being overlooked during childhood

For some participants, loneliness was discussed in relation to their family and childhood experiences. This involved stressful environments, financial strains, the emotional unavailability of their primary caregivers and other adverse experiences such as abuse and neglect. For example, P32 talked about her relationship with her father and similarly to how loneliness is described by other studies (Dahlberg, 2007), she attributed it to feeling invisible and inconsequential in his presence:

It took years for me to realise that I actually felt annihilated in his presence. I was so unacknowledged, that I almost felt like there was nobody reflecting me back and my grasp on my own existence was tenuous. I realised when I was very much an adult, that just being in his presence was enough to make me feel suicidal because I felt so unacknowledged, like somebody had rendered me invisible.

(P32)

Some participants discussed the loneliness that stemmed from having to keep secret experiences of abuse. Fears of the consequences of disclosing this and perceptions of self‐blame emotionally isolated them and made them feel that they could not reach out and ask for support. P11 said:

I was being sexually abused from the age of 9 and, and it was quite a secret thing, it was something I was keeping to myself …think it was quite lonely because I suppose it’s a lot to deal with and at the time, I didn’t really have anyone to talk to about it.

(P11)

As a child, the expectation to or discuss with people outside the household domestic violence their mother was experiencing left P37 feeling even more lonely:

I was like locked inside of the prison, you know, I couldn’t say a word I couldn’t look for help, I literally felt like I’m like decaying inside, like the living dead. You know we are forbidden to talk about this to anyone and it does make you feel lonely.

(P37)

Some participants believed that feelings of loneliness and their secret could have been identified had they not been emotionally neglected and denied the support and acknowledgement to fulfil their basic emotional needs. For P7, for example:

So, there was always that constant fear of you know something’s gonna happen. And the fear and loneliness was in my eyes, and I got to thinking that if people could just look into my eyes, they would see that there was a frightened lost lonely child in there. But nobody ever sort of asked.

(P7)

#### Being abandoned by society and those who could have helped

For many participants loneliness arose from feeling excluded and peripheral to the structures and essence of society. Many felt a lack of support from the government and local statutory services which could have helped them in their time of need. The most vulnerable and those in need of services felt that they were not being heard by the ones who could have assisted them. This relates to what has been described as ‘ethical loneliness’ as participants described feeling abandoned ‘by those who have the power over one’s life possibilities’ (Stauffer, 2015, p. 1).

Social alienation due to government cuts was seen throughout the data irrespective of age, gender etc. Thus, similar to the study of Wong (2017), loneliness was the result of insufficient care being available by the government. Social clubs and support groups, which for many were an important resource for active networking with ‘similar others’, were closed. P19 talked about the impact of two social clubs closing:

They lost their funding which was hard because I was enjoying it. I was enjoying the activities they were doing. They were doing like games, cooking in one of them. All different types of stuff… It was hard because I was enjoying it. I enjoyed seeing people.

(P19)

P3 made reference to the unmet needs of unpaid carers:

But lots of our helplines as unpaid carers has been cut through the [um] decade of austerity so, you know, you can try in vain when you need help but [um] there’s really nothing there or the organisations have, have limited their, you know, helpline to outside of 24/7.

(P3)

Other participants discussed not being heard within the mental health‐care services, not being seen as fully human and not being acknowledged beyond their psychiatric label. They described a process of dehumanisation and objectification from health‐care professionals that induced loneliness and social isolation. Similar to other studies, they recounted being treated as objects, for which the health‐care professionals did not have time to engage in meaningful dialogue (Karhe & Kaunonen, 2015). In relation to mental health care, P6 described:

If I could at least told them about my experiences and what I was going through, I wouldn’t necessarily have wanted an answer but I’d have wanted someone to listen and if someone’s listening to me, they see me, I’m not lonely anymore

(P6)

Finally, for some participants, loneliness was a result of feeling that their social identity was invalidated and undervalued. In other words, it stemmed from their role and contribution to society being undermined and not acknowledged. For example, P3 reflected on her unpaid carer role:

When I’m being sort of selfish, I think maybe a made a mistake and I should have just kept my career because they certainly don’t look after carers, they’re on their own… Because people seem to forget that the productivity coming from unpaid carers in this country saves the State about a £132 billion every year so, you know, it’s a bit of a sore point when people say you didn’t go to work

(P3)

### Lingering loneliness

For many participants, loneliness was not an ephemeral experience that could be attributed to situational causes but something that accompanied them throughout their life. It was perceived as an innate part of their identity that stemmed from a sense of otherness and its presence lingered even in the company of others. Furthermore, many described feeling trapped in a cycle of loneliness, which further perpetuated and intensified personal isolation.

#### Loneliness, identity and belonging

Similar to other studies that explored loneliness in relation to people that felt they did not quite belong in mainstream culture (e.g., Rokach, 2014), for our participants a sense of loneliness emanated from feeling ‘different’ and was inevitably ingrained in their identity. For example, P1 said:

I think [um] being autistic [um] I feel lonely quite a lot of the time [um] so I think it’s always there, I think it’s never not a part of who I am

(P1)

For some participants, being ‘different’ affected their sense of integration and belonging and emotions were tied to loneliness. P33 said in relation to mental illness:

I kind of always, I guess, had this like longing to belong or like be a part of other people’s lives where I wasn’t really. Um, so I spent kind of like most of my teen years extremely lonely.

(P33)

Being ‘different’ sometimes also meant being stigmatised and fearing or experiencing rejection. In relation to her mental illness, P39 said:

It’s like damaged goods you don’t ever, nobody ever prioritises damaged goods, you look for an apple you look for a perfect one in the supermarket you don’t look for one that has got lumps and bumps

(P39)

Many discussed the experience of a profound sense of loneliness through trying to convey their inner experiences to people who could not relate to them and the inadequacy of language in communicating with people who were not similar to them, who could not relate to their experiences and the subsequent affective ramifications. In relation to psychosis, P15 commented:

There’s a sense of loneliness that people can’t experience the same things that I’m experiencing, and people say to me, if I’m talking to somebody [um] that other people can’t see, it can be quite frustrating when people are saying, “But I can’t see that,” when it’s very real to me. And that makes you feel lonely because people aren’t experiencing the same world that you’re experiencing

(P15)

The absence of similar others, people who they can identify with, who could provide emotional support, empathic understanding and validate their identity creates what Stein and Solomon (2017) terms ‘experiential loneliness’, an emotional isolation that stems from ‘failed intersubjectivity’. P33 who was non‐binary said:

You feel very much alone and you’re very much aware that the experiences you’re having and the feelings you’re having aren’t the same as the people around you. And as much as they try and are as supportive as they can be, I don’t think that they will ever be able to fully understand. You kind of feel like if you’re trying to communicate something to them about like, something difficult about being like gay or non‐binary, they can try to understand but they won’t fully know, and I think that is a form of loneliness.

(P33)

#### Loneliness despite the presence of others

For many participants, loneliness persisted even in the presence of others. They attested to experiencing emotional isolation in a group of people, within their family or even their intimate relationship. Togetherness in some cases even intensified loneliness:

I’ve been with people and felt lonelier than when I was on my own. I don’t really know why. I guess, if you can’t relate to people, or like you don’t really feel a part of their group, then it can definitely feel lonelier to be around people.

(P25)

Loneliness was also experienced in the absence of shared understanding as P8 describes in this extract:

Going to the pub and being around people I still felt very isolated because of my HIV. And that people, there weren’t people who got where I was at, and understood [um] the position I was in, and what was going on in my mind. I felt very isolated. Even though I was surrounded by people

(P8)

However, for some participants, loneliness was not indicative of the deficient quality of their relationships. These participants described an ‘internal’ form of loneliness that kept them separated from the world, made them feel like an outsider and alienated them from others. This phenomenon is termed as existential loneliness (Nilsson et al., 2006) and is common among people affected by mental illness. P37 describes this in relation to her ex‐partner:

I used different things in those times to cover over it [loneliness] and deal with it in a different way. But eventually it would come to the surface as the relationship developed [um]…but no, I’ve never not felt this crushing emptiness and loneliness

(P37)

#### The cycle of loneliness

Most participants reported feeling trapped in a cycle of loneliness. As Killeen described loneliness ‘is a very destructive condition and it can cause a vicious downwards spiral because the more lonely one becomes the more one is isolated even further from normal society’ (1998, p. 763). They acknowledged the need to act and to ‘do something’, to reduce the aversive state of isolation, but simultaneously, loneliness had become an integral part of their life in which they felt secure. P32 described the paradox of wanting to alleviate loneliness while unwilling to step outside their comfort zone which loneliness had become:

That might sound a bit peculiar but I’ve begun to recognise that when I have the opportunity to socialise it’s almost as though I’ve turned in so much on myself, that [sighs] it’s becoming hard to turn outwards and meet other people again.

(P32)

Furthermore, many explained how loneliness creates a tendency to dwell in self‐pity, be more sensitive to rejection, think people have negative thoughts about them, be more socially isolated, withdrawn and be less trusting of the people around them. This is turn created worsening feelings of loneliness. P13 mentioned:

It makes you [um] less trusting, [um] you, you’re not very quick to let anybody in to change you, because you’re worried, it makes you worry more that you’re going to end up feeling worse than you already do

(P13)

The fear of experiencing loneliness in the future further created a barrier to enjoying the present and had an impact on the mental state of many participants. P11 talked about the fear of deteriorating health in the future:

It’s that loneliness…, that sort of scaredness feeling of, if I have ill health in the future, if there’s no‐one there and I’m going to have to try and deal with this on my own and you don’t really want to, you know, to be, to be honest.

(P11)

### The unspoken and trivialised experience of loneliness

The majority of participants discussed the difficulties of disclosing their experiences of loneliness due to the shame and stigma associated with it. The experience in their view was rendered as a taboo subject and silenced. It was confined largely to the public discourse of old age and failing health. Concurrently, participants described how, on rare occasions when loneliness was discussed, it was approached in a veneer of light‐heartedness and easy‐fix solutions. Interventions were recommended by people who did not understand their experiences.

#### The silencing of loneliness

A pivotal subtheme here was the notion of stigma, shame and self‐blame which restricted constructive discussion about loneliness. Many participants discussed the archetype of ‘the loner’ who is depicted as socially ‘defective’, inept and incapable of forming pivotal relationships to combat loneliness. P39 highlighted:

If you’re a loner there’s something wrong with you…It is not a positive word to put on someone …there must be something, because why would someone choose to be alone?

(P39)

For participants, this stereotype is based on a deficit‐model and views the individual through a lens of personal and social failings and intrinsic character flaw. Subsequently, loneliness is pathologised and viewed as an experience outside the boundaries of normalcy. For P22:

I think there was a lack of talking about loneliness as a thing that can happen to just normal people. It was always this person visibly has no social skills and you know is really profoundly like struggling in society, not this is another well‐adjusted person who has got some confidence issues and is struggling to make friends in a new context.

(P22)

The ‘tyranny of positive attitude’ which is saturated with the view that we must think positive thoughts and block out any emotions that may have a negative valence and avoid any difficult but never‐the‐less authentic emotions, further contributes to the silencing of the discussion of loneliness. This also challenges positive psychology approaches that are widely promoted as ways of dealing with loneliness (Lim et al., 2020):

We’re meant to be these very successful, bubbly, happy go lucky people who do these great things every weekend and you know, are never stressed, are never behind at work, you know, are never struggling.

(P10)

Most importantly, however, participants emphasised that in the public discourse, loneliness was perceived as an age‐specific problem. Indeed, the stereotype and trope of the lonely elderly prevails in collective consciousness leaving little room for other populations to share their experiences of loneliness. By being excluded from the loneliness discourse, many participants resorted to self‐blame for failing wider expectations. As P17 described:

They always say it’s the older people that feel lonely… They never talk about the younger generation.… I think because society expects people at that age to be married, have children, and have their lives sorted so to speak, so they don’t think about people that might be gay, they don’t think about people that might be single, they don’t think about people that might be transexual, they just think about the mainstream. And then sometimes that makes me feel guilty about being lonely or feel weird about being lonely, because I think I shouldn’t be lonely at my age, I should have lots of friends, I should have a partner. And then I think “what’s wrong with me? Why do I not have these things?”

(P17)

The stigma attached to loneliness dissuaded many participants from seeking support from services. Indeed, society’s intolerance towards individuals who experience loneliness acted as a barrier to help seeking. In reference to accessing help for loneliness P5 mentioned:

I would have really benefitted from it at the time [support] I don’t know if I would have accepted it if anybody had said it because I also think there’s that kind of, you don’t wanna be stigmatised

(P5)

#### The trivialisation of loneliness

Many participants asserted that when loneliness appears in public and private discourse, it is trivialised and its seriousness is diminished. The consequences it might have for a person’s quality of life and mental health is downplayed and the reality, nuances and causes of the experience are oversimplified and even ignored. Moreover, the legitimacy and severity of loneliness is questioned and the discourse around it imply that it may be the fault of the individual. People are assigned blame for not being proactive and for not taking steps to make positive changes. Thus, individual agency was presented to many participants as an effective approach to managing loneliness. This is illustrated by the following extracts:

People just go, “Oh, loneliness, you just need a fellow,” or “Loneliness, oh get a dog,” or, it’s just people minimalize it so that it’s, loneliness leads to so much more than just being lonely… It can lead to all sorts of things that people don’t necessarily think would start from loneliness.

(P13)

I don’t think people would suggest you go to therapy for feeling lonely. They wouldn’t suggest professional help for feeling lonely. Um, they’d just kind of be like, “Oh, just call up a friend. Just go hang out with a friend.” Um, but sometimes loneliness goes a lot deeper than that.

(P33)

Since the essence of loneliness is oversimplified, the services and advice provided by the ‘experts’ for people who experience loneliness are unsophisticated and only superficially approach the problem. P5 shared her experience of being given ‘by the book’ advice from the health visitor that was not tailored to her circumstances or needs, when being a new mother and experiencing acute loneliness:

You get all the health visitors come round and all that kind of stuff and I always felt like they were just saying okay yeah but you can just fix that, you know, can you do this? can you that? can’t you do such things like it’s easy to, to kind of just get on with things and not feel isolated.

(P5)

Indicatively, participants discussed support from services that aimed to broaden people’s social networks and therefore to tackle social isolation (quantity), whilst they felt that they lacked a close intimate attachment to another person (quality). Thus, strategies between social isolation and emotional loneliness were interchangeably used indicating an insufficient understanding of the differences between the two. Many participants suggested that genuine attachments could not be formed in loneliness support groups that just come together for social reasons since they are not based on common interests between the individuals but solely driven by despair and a desire to connect. For P2:

I just feel like in that [support group for loneliness] group other people will just be maybe desperate for friendships…like some people are lonely, they’re just happy to be friends with anyone [um] and obviously that’s not what I want. I want friendships to be genuine…you need to have a rapport, a rapport and [um] genuine fondness for each other and a likeness with [um] appreciation for each other’s company

(P2)

## DISCUSSION

A strength of the research is the unusual heterogeneity of our sample which allowed us to explore a range of experiences and views and identify common features of loneliness linked to a diverse population. It highlighted the complexity and nuances of loneliness among adults and the need to avoid extrapolating the findings of research by age categories to other populations. The loneliness experienced by participants did not necessarily conform to one‐dimensional conceptualisations of loneliness and were more multifaceted than a perceived discrepancy between desired and achieved level of social relations which is the basis of the widely used contemporary definition. The experiences of loneliness were highly subjective, linked to the absence of a significant someone, thus touching the interpersonal realm but also associated with participants’ roles and identity within society. Indeed, for many participants, loneliness arose from inadequate integration and alienation within society, through insufficient care and community resources, stigmatisation and feelings of being abandoned by those in power. Their devalued identity rendered them invisible and, similarly to the loneliness articulated by Stauffer (2015) they felt dehumanised by being pacified, not being heard and unjustly treated. The main approaches examining the causes of loneliness have focussed on individual level characteristics and determinants that predispose people to loneliness. However, De Jong Gierveld (1998) highlighted the need to explore the social and economic circumstances contributing to loneliness. Our study indicated that a wide range of macro‐level factors generate feelings of loneliness.

Our study provides a nuanced understanding of the reasons people may struggle to talk about loneliness. Our participants suggested that loneliness and the causes that lead to loneliness are often shrouded in shame, trivialised and ignored. Consistent with findings from previous studies (e.g. Franklin et al., 2019), our research suggests that people who experience loneliness may be reluctant to discuss the subject for fear of being stigmatised. Indeed, the literature indicates that lonely people’s fear of negative evaluation may be justified. Studies suggest people tend to attribute more negative characteristics to those who they deem lonely. Indicatively, Lau and Gruen (1992) asked college students to provide their impression of a hypothetical lonely and non‐lonely peer. Findings revealed that lonely peers were rated as less competent, warm, adjusted and likeable and participants expressed less compassion towards them in comparison to their non‐lonely peers. Although, more recently, Kerr and Stanley (2021) suggest these negative characteristics are attributed to individuals only when loneliness is perceived as a volitional behaviour, the overall findings illustrate that lonely people are disparaged. The stigma attached to loneliness, as is the case for mental illness, obviates the move towards recognising loneliness and later seeking service support. Due to this stigma, we acknowledge that the voices of those who feel the need to shy away from the label of ‘loner’ might not have been included in the study (Pinquart & Sorensen, 2001). We suggest that further research is needed to consider the interplay between wider structures and loneliness and tease out the resonance of our themes for specific groups.

Our study also highlights the need to normalise the experience of loneliness in the public discourse and promote openness amongst those who are affected by it. Existentialist theory of loneliness sees the phenomenon as a painful but also an intrinsic human condition and mandatory for personal growth (McGraw, 1995). For Moustakas, loneliness ‘is an experience of being human which enables the individual to sustain, extend and deepen his humanity’ (2016, p. ix). From a biological perspective, Cacioppo et al. (2006) conceived loneliness as an aversive signal, nevertheless, similar to hunger and thirst, that protects people from social isolation and contributes to the maintenance or repair of meaningful social connections. However, our analysis demonstrates how people who identify as lonely are perceived as distinct, different, inferior and weak‐willed whilst the multiplicity of variables that contribute to loneliness are not acknowledged. Society’s role in creating loneliness is downplayed and causes are attributed to the individual. Indeed, in the zeitgeist of positive thinking and self‐control, loneliness can only be perceived through the lens of personal failing (Joffe & Staerklé, 2007). Simultaneously, an overwhelming sense of responsibility is bestowed on the individual to overcome loneliness, which is caused by factors outside their control. Like many issues related to mental health, loneliness has been discussed in the public discourse solely in the realm of private life and personal control. Furthermore, as a result of the enduring stereotype of loneliness in old age, which monopolises the discourse in relation to loneliness, the loneliest are left to dwell in their loneliness.

It has been suggested that loneliness in older age is overestimated and therefore perceived to be a greater problem than is in reality (Dykstra, 2009). A recent study (Barreto et al., 2021), based on the frequency of loneliness of 46,054 participants aged 16–99 living across 237 countries, reveals that young people is the group that feel loneliest. Thus, 40% of 16–24‐year‐olds reported experiencing loneliness often or very often, compared with only 27% of those over the age of 75 years. Therefore, the stigma of the phenomenon can be challenged by reframing public discourse. This can be done by removing the blame from the person and abstaining from approaching loneliness as a default deficit within the individual or a consequence of individual action or choice. There is a need to approach loneliness as a recurring condition of human social life that transcends age, gender and geography.

Whilst it is important to highlight that loneliness can be encountered in anyone and everyone, with minimised and reframed pathological notions of their experience, it is equally important not to minimise its severe consequences and the need to seek support. Loneliness can be an issue of serious concern when left unattended and when it settles long enough to create a persistent self‐enforcing loop of negative thoughts, sensations and behaviours (Cacioppo & Patrick, 2008).

Many participants described a form of prolonged loneliness that was persistent since childhood; nevertheless, they were not living alone, they were integrated in society and did not necessarily feel a lack of satisfying relationships. They described a loneliness attributed to the lingering rejection of their identity and differentness. Therefore, this research expands the criteria that should be put forward to reveal the factors facilitating prolonged loneliness.

Most participants longed for emotional togetherness, and the presence of social contact could not combat the persistent feeling of loneliness. However, interventions that aim to decrease social isolation have tended to be studied and evaluated in conjunction with the ones targeting loneliness.

The present study identifies several directions for future research and interventions. The findings highlight a neglected topic related to the untimely death of a loved one, loneliness and lack of emotional support for the unexpectedly bereaved. Future research could further explore the social needs of bereaved people who do not fit commonly held stereotypes. Furthermore, the results make a case for future research to study loneliness in relation to the position and the relationship one has with their community. Instead of exploring the phenomenon through the lens of ‘personal failing’ and ‘deficit’, research should identify government mechanisms and social infrastructures that isolate an individual or a social group. Our study indicates that loneliness is experienced in all age groups including childhood which further supports the argument that loneliness should be studied through a life course approach and longitudinally. Finally, our findings indicate that loneliness is a socially stigmatised state of being, therefore it is important to explore the factors that contribute to its stigmatisation and to ensure interventions aiming to ameliorate it do not unintentionally further perpetuate shame and misconceptions around it.

## CONCLUSIONS

Our study contributes to understanding how loneliness is experienced by a wide range of adults who do not necessarily fit into a homogenous and unitary group. The findings suggest that loneliness may stem from unfulfilled interpersonal social needs but also from a societal undermining and invalidation of people’s social identity. Unmet care and support need ignored by those with the power to help left participants feeling unheard, in turn perpetuating feelings of abandonment and social alienation. Furthermore, the stigmatisation of loneliness left the most vulnerable to endure the phenomenon in silence. These findings should be considered when developing interventions that aim to ameliorate loneliness.

## AUTHOR CONTRIBUTIONS

**Melina Aikaterini Malli**: Conceptualisation (Supporting); Data curation (Lead); Formal analysis (Lead); Methodology (Equal); Writing—original draft (Lead); Writing—review & editing (Equal). **Sara Ryan**: Conceptualisation (Lead); Formal analysis (Equal); Funding acquisition (Lead); Methodology (Equal); Validation (Lead); Writing—original draft (Equal); Writing—review & editing (Equal). **Jane Maddison**: Formal analysis (Supporting); Writing—original draft (Supporting); Writing—review & editing (Supporting). **Kalpa Kharicha**: Writing—original draft (Supporting); Writing—review & editing (Supporting).

## Data Availability

The data that support the findings of this study are depostited in the Medical Sociology and Health Experiences Research Group, University of Oxford. Please contact Hergadmin@phc.ox.ac.uk for further information.
